# Epistemic cognition in medical education: a literature review

**DOI:** 10.5116/ijme.5849.bfce

**Published:** 2017-01-07

**Authors:** Jennifer L. Eastwood, Elysa Koppelman-White, Misa Mi, Jason Adam Wasserman, Ernest F. Krug III, Barbara Joyce

**Affiliations:** 1 Department of Anatomy and Cell Biology, Burrell College of Osteopathic Medicine, Las Cruces, New Mexico, USA; 2 Department of Philosophy, Oakland University, Rochester, Michigan, USA; 3 Department of Biomedical Sciences, Oakland University William Beaumont School of Medicine, Rochester, Michigan, USA

**Keywords:** Epistemic cognition, epistemology, uncertainty, humanism, medical education

## Abstract

**Objective:**

To review the research literature on epistemic cognition in medical education.

**Methods:**

We conducted database searches using keywords related to epistemic cognition and medical education or practice. In duplicate, authors selected and reviewed empirical studies with a central focus on epistemic cognition and participant samples including medical students or physicians. Independent thematic analysis and consensus procedures were used to identify major findings about epistemic cognition and implications for research and medical education.

**Results:**

Twenty-seven articles were selected. Themes from the findings of selected studies included developmental frameworks of epistemic cognition revealing simple epistemological positions of medical learners, increasing epistemological sophistication with experience, relationships between epistemic cognition and context, patterns in epistemic orientations to clinical practice, and reactions to ambiguity and uncertainty. Many studies identified the need for new instruments and methodologies to study epistemic cognition in medical education settings and its relationship to clinical outcomes. Relationships between epistemological beliefs and humanistic patient care and influences of medical education practices were commonly cited implications for medical education.

**Conclusions:**

Epistemic cognition is conceptualized and operationalized in a variety of ways in the medical research literature. Advancing theoretical frameworks and developing new methodological approaches to examine epistemic cognition are important areas for future research. Also, examination of the relationship between the contexts of medical learning and practice and epistemic cognition has potential for improving medical education. This work also establishes a need for further investigation into the implications of epistemic cognition for humanistic orientations and ultimately for patient care.

## Introduction

As contemporary medical curricula increasingly emphasize disciplinary integration, cultural competence, evidence-based medicine, and research, learners are required to draw upon diverse ways of knowing and learning. They must be able to evaluate and compare various sources of evidence, reflect upon their actions, and consider different perspectives, belief systems, and roles in the healthcare setting. Medical learners are expected to integrate knowledge from different disciplines and make decisions in the midst of ambiguity. Additionally, increased emphasis on research positions learners both as creators and consumers of knowledge and confronts them with the task of evaluating the authority and validity of information.^1^ As learners tackle these cognitive tasks, they activate personal theories of knowledge and knowing, which influence how they reason, make decisions, and make meaning out of their experiences.^2^ Thus it is important to consider how such theories relate to medical education and practice.

The study of epistemology, defined as theory or discourse about knowledge, includes a diverse body of scholarship in various disciplines including psychology, philosophy, and education.^2^ Epistemology focuses on what knowledge is as well as how it is produced, acquired, and justified.^2^^,^^3^^,^^4^ While terminology describing this domain of work varies and has nuanced meanings,^2^^,^^5^ we use the term, epistemic cognition, described by Green, Sandoval and Braten as concerning “how people acquire, understand, justify, change, and use knowledge in formal and informal settings”. Other terms describing this area of research include personal epistemology,^6^^,^^7^ epistemological beliefs,^8^^,^^9^ epistemological resources,^10^ and reflective judgment.^11^

To clarify and elaborate specific components of epistemic cognition, Hofer and Pintrich developed a model based upon their review and synthesis of research in this area. Dimensions of the model include certainty of knowledge (absolute to relative), simplicity of knowledge (unambiguous to complex and interrelated), source of knowledge (authority-based to constructed by the knower) and justification of knowledge (directly observed or received to critically evaluated).^12^ A large contingent of research describes epistemic cognition as developmental in nature, applying a simple to sophisticated continuum. Ideally, individuals progress over time toward conceptions of knowledge as uncertain, context-dependent, constructed, and critically evaluated, but also integrating personal values and relational ways of knowing.^12^^,^^13^^,^^14^ With her work on epistemological beliefs, Schommer challenged developmental stage models and described epistemic cognition as a set of independent beliefs about knowledge and knowing.^8^ Some more recent research focuses on the contextual nature of epistemic cognition and incorporates goals, values, and virtues.^2^^,^^5^^,^^10^

Considering that medical education and practice demand sophisticated ways of thinking, it is important to consider what is known about learners’ and practitioners’ theories of knowledge. Thus, the aim of this study is to review the research literature to identify what is currently known about epistemic cognition in medical education as well as its implications for teaching and research.

## Methods

Following the recommendations of Cook and West,^15^our goal is to analyze and synthesize the research literature on epistemic cognition as a means to identify (1) what is known about epistemic cognition in medical education and practice, (2) how it informs medical education, and (3) what areas are in need of additional research. No ethical approval was required for this study, as it is a review of literature.

### Search process

Comprehensive literature search strategies were formulated for seven databases, including Ovid MEDLINE, SCOPUS, Web of Science, EMBASE, ERIC, CINAHL, and PsycINFO. Two librarians constructed and peer-reviewed search strategies which included combinations of index terms (e.g., MeSH terms) and keywords to capture the complex concept of epistemic cognition. These terms and keywords included: epistemology, knowledge, medical philosophy, uncertainty, ambiguity, cognitive flexibility, situational awareness, reflective judgment, intellectual development, self-reflection, narrative, competency, reflective practice, episteme*, epistomolog*, uncertaint*, certain*, and ambigu*. These terms were combined with terms related to medical education: physicians, internship, residency, medical students, clinicians, fellows, interns, medical education, and medical schools. Unique indexing terms for each database were also identified and searched. References of selected articles were hand-searched to identify additional relevant articles. Search results from each database were imported to a bibliographic management system, and duplicate references were removed.

Two authors independently screened titles to exclude any articles that were not: 1) peer-reviewed, 2) empirical, 3) focused on physicians or medical trainees as primary participants, and 4) focused on epistemic cognition as the central object of inquiry. This process was repeated in more depth when the same two authors independently excluded additional articles based on review of abstracts, and then again with full text articles.  At these last two stages of the selection process, a third author was brought in to independently evaluate articles on which the two screeners disagreed. 

### Data abstraction and analysis

Categories for data abstraction included study characteristics (date of publication and journal), information about participants (sample size, role in medical field, specialty, and nationality), research methods (methodology, data source, instruments, analytic techniques, and core measures), major findings, and implications identified by the authors. Analysis of data in the categories of major findings and implications involved qualitative thematic analysis.^16^ For each article, two researchers independently conducted iterative processes of reading text, coding central ideas as themes, and refining those themes to capture and interpret the meaning of the text.^16^^,^^17^

For all categories, a spreadsheet was used to facilitate coding by multiple researchers. Two authors independently coded each full text article to abstract data for each category. Coding pairs met to resolve discrepancies before entering final data into a database. For each category, themes and patterns emerging from the data were identified, clarified, and refined among pairs and then reviewed by all authors.^18^

A quality assessment scale^19^ was adapted to assess the methodological constitution of selected studies. However, because of substantial variation in methodology and discipline, we utilized this scale to provide a qualitative description rather than numerically scoring article quality.

## Results

### Trial flow

**Figure 1:**
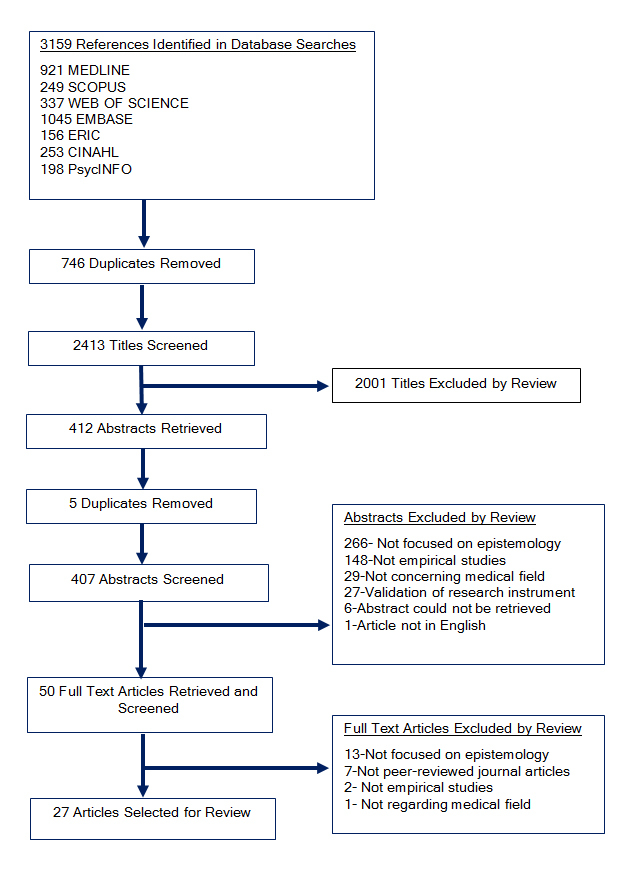
Summary of study selection process

Our database searches yielded 2413 titles after removal of duplicates, of which 407 were considered potentially relevant based on title. Of the 407 abstracts reviewed, 50 articles were retained for full text review. Before full text review, the research team revised selection criteria to include only studies that systematically collected and analyzed data to evaluate a research question, and articles that include explicit discussion of orientation toward knowledge or epistemology. For example, studies reporting scores from instruments that measure ambiguity tolerance without exploring epistemological aspects of the measure were excluded. As a result, final selection for the review includes 27 studies (Figure 1).

### Article quality

The diverse ways of conceptualizing epistemology naturally produce a great deal of variation in methodological approaches. There were some observable trends, however, with studies targeting clinical uncertainty tending to be quantitative, while qualitative studies tended to explore wider meanings of ways of knowing by medical students. Sample compositions ranged from those drawn randomly from within an institution to those selected on the basis of having registered for a particular course or program.^20^ Only one study attempted to stratify the selection of respondents by factors such as specialty and role, but the resulting numbers in each category were quite small.^21^ The sophistication of the analyses ranged from basic descriptive statistics to more sophisticated linear modeling techniques.  On the qualitative side, several studies used pre-fabricated coding schemes, while others employed a grounded-theory-like coding scheme, coding narrative data de novo.  However, only one study^22^ utilized an iterative process of theoretical sampling to achieve a “saturation” point where no new themes emerge, suggesting the terrain has been circumscribed.^23^^,^^24^ While the narrow construct of “tolerance of ambiguity” appeared a popular solution for wrangling the difficult notion of epistemology, broader understandings of how medical students think remained poorly defined.

### Study characteristics

The majority of the studies (20/27) were published since the year 2000. Disciplinary affiliations of the journals in which these studies appear were concentrated within education (mostly medical education) and sociology. Six of the studies were published in family medicine journals, most of which focused on ambiguity and uncertainty. Most studies reported small sample sizes; about half had 40 or fewer participants, and most included qualitative approaches (16 qualitative and 3 mixed methods). The samples of most studies (16) included medical students, but about half also included physicians and residents. The majority of the studies were conducted in the United States (11) and Western Europe (11), but our review represents research conducted in twelve countries (Appendix 1).

### Themes in major findings of studies

#### Developmental models

Four of the studies applied developmental schemes of epistemic cognition, including Perry’s epistemological positions^13^ and the Reflective Judgment Model.^11^^,^^25^^,^^26^^,^^27^^,^^28^ In their samples of medical students and physicians, authors identified relatively simple conceptions, including justification of knowledge through invoking authority or personal experience, views of knowledge as certain and concrete, and the expectation that solutions to problems will be logical or obvious. More sophisticated views of participants included justification based upon multiple considerations, such as the weight of evidence and explanatory value of interpretations, and recognition of patient perspectives in complex problems.^25^^,^^27^ 

Studies applying Perry’s epistemological positions^13^ found most medical students and first year residents to be dualistic thinkers, espousing black-and-white views of the world in which authorities have the answers and learners receive them. Second and third year residents were mostly classified as being in “multiplicity,” a state of being able to recognize uncertainty, accept that some problems do not have “correct” answers, and recognize that multiple opinions can be equally valid.^26^^,^^28^ Additionally, medical students were found to be more dualistic as compared to psychology students who were more relativistic. The authors attributed those findings to differences in training.^26^

Applying the Reflective Judgment Model,^11^ two studies identified most second year medical students, first year general practice trainees, and trainers as pre-reflective and quasi-reflective.^25^^,^^27^ In pre-reflective stages, individuals perceive knowledge as concrete and observable, uncertainty as temporary, and truth as passed down from authorities.  In quasi-reflective stages, individuals tend to view all ideas as equally valid, but begin to recognize that knowledge is contextual and relative.^29^ Epistemic cognition commonly varied with regard to different disciplines, even within the same individual, where humanistic elements of medicine were considered more complex and uncertain and biomedical sciences were seen as fact-based with known or knowable answers.^25^^,^^27^

### Changes in epistemic cognition with experience

Several studies indicated that epistemic cognition changes over time and with experience using retrospective reflections of participants or cross-sectional studies. For example, awareness of uncertainty in clinical situations increases over time.^30^^,^^31^^,^^32^ Lingard et al. found that medical students adopted physicians’ ways of speaking about uncertainty with greater confidence,^33^ and Nevalainen et al. noted that students learn to recognize uncertainty as part of medicine and to accept that their knowledge is incomplete.^20^ Reflecting on their experience in medical school, second-year medical students described changes in their epistemic views, especially in certainty of knowledge. In considering humanistic elements of medicine, such as understanding patients’ experiences, they began to question black and white conceptions of knowledge and recognize the possibility for error.^25^ Furthermore, Gordon et al. conclude that the process of assimilation, in which students reflect upon meanings of experiences and incorporate them into their knowledge structures, is learned over time.^34^  Three studies pointed to the transition from medical student to physician as a critical time for assimilation of knowledge and values, gaining awareness of and learning to manage uncertainty, and understanding complexity of patients’ stories.^32^^,^^34^^,^^35^

#### Epistemic orientations to clinical practice

Three studies focused on orientations to clinical practice including biomedical and biopsychosocial epistemologies or cure and care orientations.^30^^,^^36^^,^^37^ These orientations were described as conceptual resources for managing uncertainty^30^^,^^36^ or attitudes guiding interactions with patients.^37^ Biomedical epistemologies are characterized by dualism of the mind and body, reduction of health to its biological parts, primacy of biological data, and disease as disruption of normal physiology. Biopsychosocial epistemologies are characterized by a holistic view of the mind and body, disease as a biological, social, and psychological phenomenon, and the clinical approach as integrating all dimensions of illness.^30^ Evans and Trotter found a positive relationship between biomedical epistemology and stress reaction to uncertainty in physicians.^30^ Examining third-year medical students, Evans et al. found that biomedical epistemology was related to higher stress reactions at the end, but not the beginning of the first clinical year. The authors suggest that significant epistemological development occurs upon entering the clinical setting and that a more integrative epistemology may help students cope more effectively with uncertainty.^36^

In studies of orientations to clinical practice, no gender differences were found in cure-versus-care orientations among third-year medical students^37^ or biomedical versus biopsychosocial orientations in resident and non-resident physicians.^30^^,^^36^ Similarly, no significant differences in medical epistemologies were found among different specialties.^30^ However, a group of Dutch medical students were found to be more care-oriented than Belgian students. According to the authors, communication classes in the Dutch program, cultural differences, or different phases of training may have contributed to the difference.^37^

De Valck et al. challenged the conception of medical epistemology as a continuum spanning from a cure attitude (similar to biomedical epistemology) to a care attitude (similar to the biopsychosocial epistemology). Using medical student responses to the Ideal Physician Questionnaire,^38^ factor analysis supported the existence of separate “instrumental” and “affective” domains, which allowed for four separate profiles instead of dichotomous cure and care categories. The authors suggest that adopting positive views toward an instrumental approach to medicine in order to “cure” disease does not necessitate a detached approach to relating to patients. Focusing on both instrumental and empathic aspects of medicine can promote “integration of a humane attitude while maintaining professional competence in diagnostic reasoning and decision-making”.^37^

### Role of context

A common theme among the studies was the importance of the educational or clinical context in epistemic cognition, particularly in the adoption or expression of particular views. Discussion of contextualized cases elicited recognition of uncertainty^39^ and examination of varying contexts elicited different epistemological views or approaches to understanding.^40^ Contextual factors of the clinical or educational environment or of individual patient cases, such as severity of an illness, risk involved in decision-making, and the patient’s desire for certainty are thought to influence physicians’ reactions to uncertainty and perception of “gut feelings,” implicit knowledge characterized by feelings of alarm or reassurance.^22^^,^^28^ Also, socialization through pre-medical coursework and public media may influence entering medical students to be more aware of uncertainty in medicine.^41^ Conversely, reliance on technology and the fast pace of scientific knowledge development may foster desire for structure, intolerance of ambiguity, and views of knowledge as certain.^25^^,^^44^ Additionally, methods of instruction and assessment may influence students’ epistemic approach to be more focused on memorization of isolated facts.^45^

Experience with particular contexts during medical training appears to facilitate change in epistemic cognition. For example, Roex et al. found that greater first-hand knowledge of the clinical context activated epistemological beliefs, allowing trainers and trainees to be more aware and critical of other perspectives.^27^ Similarly, once a student becomes familiar with the context of medical practice, he or she may adopt a more emic or insider perspective to meet the demands of the social context.^46^

Several studies discussed how epistemic cognition is influenced by context as students respond to particular objectives or expectations. Some discussed dispositions such as “thinking as a student,” having a “professional orientation,” and learning as a means to a final goal, where students only appreciate immediately applicable information, focus on instruction and evaluation, and seek to minimize anxiety.^26^^,^^33^^,^^39^^,^^46^ For example, medical students sought fact-based education on cultural diversity that they could use “to promote a sense of professional security” and avoid the discomfort of uncertainty^21^ and residents “pathologized” the concept of denial as a “disease object” requiring treatment in terminally ill patients, potentially as a way to manage uncertainty at the end of life.^47^ Additionally, because of a lack of training in epidemiology combined with little time for research, physicians struggled to integrate evidence-based medicine into practice and therefore often preferred pre-digested information as opposed to critically examining sources.^48^

### Reactions to ambiguity and uncertainty

About half of the selected studies are focused on uncertainty and ambiguity. While many other studies of uncertainty or ambiguity in medicine were identified in our search process, only studies that explicitly positioned these concepts as epistemological were selected for this review. However, our review found that even the selected studies tended to focus on reactions to uncertainty and/or ambiguity while leaving the nature of epistemic cognition largely unexplored.

The studies described below examined students’ and physicians’ “intolerance” of ambiguity, or perception of ambiguous situations as threatening.^49^ While DeForge & Sobal found no gender differences in ambiguity tolerance among family practice residents,^42^ Geller, Faden, & Levine found that females were more tolerant of ambiguity than males.^43^  Weissenstein, Ligges, Brouwer, Marschall, and Friederichs found females were slightly more tolerant of social conflicts and insoluble problems, while males were more open to new experiences.^44^ No significant differences in views of uncertainty or ambiguity tolerance were found among students in different years of medical school or between physicians and medical students,^41^^,^^43^^,^^44^ but in one study, first-year family practice residents were found to be less tolerant of ambiguity than residents in their second and third years.^42^ While no difference in ambiguity tolerance was found between community-based and university-based residents,^42^ dual degree MD/MBA students were found to have higher tolerance of ambiguity than traditional medical students.^40^ One study found no difference in ambiguity tolerance between medical students with different specialty preferences,^44^ although another study found medical students with specialty choices in psychiatry were more tolerant of ambiguity than those interested in surgery.^43^ Additionally, Geller et al. found that medical students with less judgmental views toward alcoholics were more tolerant of ambiguity.^43^

Several studies identified stress and anxiety as common reactions to uncertainty. Two studies found that physicians’ stress due to uncertainty was significantly higher in females than males.^30^^,^^31^ Evans et al. found no significant difference in anxiety due to uncertainty in male and female medical students, but found that physicians with ten or more years of post-residency practice had significantly lower anxiety with uncertainty than those with less experience.^36^ Gerrity et al. also found that physicians with more time in practice had lower stress associated with uncertainty.^31^ Differences in stress reactions associated with uncertainty were seen among specialties, with generalists having higher levels of stress than surgeons and subspecialists ^31^ and pediatricians having higher stress levels than family medicine physicians.^30^ While patterns in reactions to ambiguity and uncertainty were found among different groups, Gerrity et al. noted that levels of anxiety with uncertainty varied within individuals for different situational contexts. For example, a physician noted that "the emotional response to uncertainty is a function of the consequence of being wrong”.^31^ Additionally, Evans et al.^24^ and Gerrity et al.^31^ identified the transition from preclinical to clinical education as a critical time for developing tolerance for uncertainty and learning to manage associated stress.

### Themes in identified implications for future research

Within the discussion of their findings, several authors identify the need for new methodologies to study epistemology in medical education and practice. Longitudinal studies could show more conclusively how epistemic cognition changes over time and in different phases of education and practice.^30^^,^^31^^,^^41^^,^^42^  Developing new, psychometrically valid instruments could allow researchers to more accurately study epistemological phenomena, such as the ability to discriminate between tolerance of ambiguity and uncertainty.^43^ Mixed-methods studies could build upon and scale up qualitative work on reactions to uncertainty,^35^ and ethnographic and narrative methods could better elucidate nuances of epistemic cognition as enacted in naturalistic settings.^25^^,^^27^^,^^41^ Additionally, multidisciplinary research may be useful to examine internal and external factors from different perspectives.^31^

Authors also discuss the need to identify and examine other variables related to epistemology such as aspects of professional identity formation.^25^^,^^30^^,^^31^ Some call for research on whether epistemic cognition can be changed through education,^41^^,^^43^^,^^44^ and several cite the need for research on the relationships among education on epistemology, physician behavior, and clinical outcomes.^22^^,^^27^^,^^31^^,^^36^^,^^43^

### Themes in identified implications for medical education

Several studies discuss implicit messages in medical education that negatively influence trainees’ epistemic cognition and thus humanistic patient care. Dogra et al. found that medical students tend to seek a more simplistic conception of cultural diversity and pressure faculty to provide facts and clear-cut answers in cultural diversity curricula.^21^ The authors argue that integration of humanities in medical education can promote “a tolerance for ambiguity, provide a basis for the reconciliation of competing values, and foster the ability to discern the narrative thread in the setting of illness”. Lingard et al. inferred that medical students’ conceptions of credible sources of information are influenced by implicit messages that patient accounts are unreliable, thus preserving physicians’ authority.^33^ Approaches used to teach evidence-based medicine, de Camargo argues, often fail "to acknowledge the extensive social, economic and even political roots of the dilemmas faced by doctors”.^48^ This is consistent with other authors who have argued that the prominent discourse of evidence-based medicine prioritizes knowledge from experimental research over experiential knowledge; the concept of knowledge translation overshadows views of medical knowledge as socially-negotiated, value-laden, or built from experience.^50^^,^^51^

Several studies suggest specific pedagogical approaches to enhance epistemological development.  Among these are integrating first-hand patient experience early in the curriculum to encourage understanding and management of uncertainty,^21^^,^^32^ incorporating, problem-based learning to enhance relativistic epistemology,^26^ and providing students with tools and resources for judging and interpreting medical information.^48^ Additionally,  Stolper et al. argue that tacit “gut feelings” should deliberately be explored in the curriculum as ways of knowing.^22^

Several authors in our data set advocate for reflective or narrative educational methods to enhance coping with uncertainty, understanding the patient’s perspective, and other aspects of epistemological development.^20^^,^^52^  Three studies cite the need for an explicit and reflective approach to teaching about epistemological aspects of medicine, such as uncertainty, subjectivity, and authority in light of implicit messages in the medical context.^30^^,^^34^^,^^36^ Evans and Trotter argue that epistemology should be discussed in context, where physicians or trainees are able to “think through the clinical implications that accompany an epistemological commitment”.^30^ 

## Discussion

Through review of the literature on epistemic cognition in medical education, we found that analytical procedures, sample sizes, disciplines of journals, and geographical locations of studies varied considerably. The sample included a high proportion of qualitative and mixed methods research, which may relate to increased attention to the value of qualitative research as a means to explore complex issues in medical education and practice.^53^   This interest may increase the attractiveness of studying epistemology by making that work more publishable. 

The studies we reviewed found that although uncertainty in medicine was widely recognized, medical students and physicians had relatively simple epistemic positions. However, epistemic cognition was found to relate to the context of the educational or clinical environment and generally to increase in sophistication over time. Biomedical and biopsychosocial epistemologies were found to correlate with anxiety associated with uncertainty in the clinical environment, and approaches to learning were found to relate to epistemic cognition and context of education. Additionally, reactions to ambiguity and uncertainty were shown to be nuanced among specialties, gender, and years in practice.

### Limitations and strengths

Despite a thorough search process, the variability in terminology used to describe epistemology as well as the limitation to English language studies may have prevented us from identifying some relevant studies. While the diversity of approaches to research in our selection made systematic evaluation difficult, the interdisciplinary composition of our team allowed us to capture and synthesize various disciplinary perspectives. Furthermore, review of this burgeoning area of research is important for the ongoing development of inquiry into epistemic cognition in medical education and practice to understand what is already known and what remains to be discovered.

### Relation of studies to frameworks of epistemic cognition

The findings of studies included in our review were largely consistent with developmental schemes of epistemology,^11^^,^^13^^,^^14^^,^^29^ however, selected studies also found variability within individuals, consistent with Schommer’s model of epistemic cognition as a system of independent beliefs.^8^^,^^9^ From our analysis, it is also clear that in many instances, epistemological views of medical students and physicians relate to practical goals and perceived demands of the social and institutional context. Hammer and Elby’s framework, in which fine-grained “epistemological resources” are triggered by specific contexts,^10^^,^^54^ may help to explain these findings.

It is important to note that developmental frameworks and associated methods of measurement applied in selected studies classify and label beliefs with value-laden terms like “simplistic,” “sophisticated,” “dualistic,” and “holistic.” When examining epistemic cognition, researchers should consider whether results interpreted as simplistic or immature epistemological views may be interpreted in other ways. For example, assuming a higher level of certainty than truly exists may serve to protect a student’s sense of competence in medical training or reduce tension in interactions between a clinician and patient.^55^ Projecting certainty and authority is expected of physicians,^31^ who may view uncertainty as a threat to their societal status, self-image, and relationship with patients, sometimes leading to denial of uncertainty.^56^ Additionally, instructional methods in medical education may encourage students to view science as facts in which correct answers to problems can always be found.^57^ Because epistemic cognition clearly can change in relation to experience and interactions, considering the social context of medicine will be important in designing interventions to advance epistemic cognition.

### Relation of studies to humanistic medicine

Our review suggests that epistemic cognition plays an important role in embracing a humanistic approach to medicine. Education from a humanist perspective guides students to appreciate their roles and responsibilities relative to others, establish moral values, and create meaning from experiences.^58^  Furthermore, it is the responsibility of educators to encourage critical reflection on injustice and personal bias^58^^,^^59^ and provide opportunities for students to understand illness from the perspective of the patient.^60^ Achieving the goals of humanistic medical education assumes that the student is able to critically evaluate situations and understand knowledge as relative, contextual, and socially constructed.^58^ Thus it is plausible that physicians with more simplistic epistemic dispositions may have difficulty understanding patients’ perspectives, relinquishing authority to engage in shared decision-making, and applying knowledge and values relative to particular contexts.^61^

In line with a humanistic approach to medical education, several studies suggest that giving consideration to the social and emotional aspects of the patient experience may bring about awareness of uncertainty in medicine.^25^ Uncertainty is inherent to the processes of diagnosis and treatment, and a relativistic conception of knowledge is needed to understand that a patient’s evaluation of a situation may be different from that of the physician. Acceptance of uncertainty also may help physicians to engage in controversial discussions and to address stigmas.^43^ However, beliefs about authorities as the source of knowledge potentially hinder synthesis of knowledge from different sources and perspectives, including the patient’s.^25^ Also, beliefs about subjectivity of knowledge may help students better understand different concerns, values, and reactions of patients, such as the meaning of “a good death”.^47^

### Epistemological approach to uncertainty and ambiguity

While the connections between epistemic cognition and tolerance of ambiguity and uncertainty are not well understood, it is assumed that epistemic cognition influences such dispositions and behavior. Evans et al. assert that “primary care physicians should give strong consideration to how their epistemological commitment influences their affective and behavioral reactions to uncertainty".^30^ Biopsychosocial epistemologies have been found to relate to lower anxiety with uncertainty, but specific elements of epistemic cognition that influence physicians to be more comfortable with uncertainty appear unknown. Also, although popular perception suggests that evidence based medicine mitigates uncertainty, Timmermans et al. suggest that it instead brings the existence of uncertainty to the fore by highlighting limitations of evidence based medicine in clinical practice.^62^ Thus an epistemological approach to uncertainty in medicine has great potential to advance physicians’ integration of information from various sources, particularly for complex patient cases.

### Implications for medical education

Approaches to teaching that address epistemological themes explicitly and engage students in reflection through contextualized examples of those themes have been found to promote sophisticated epistemic beliefs about science^63^ and have potential to enhance epistemic cognition in relation to both scientific and humanistic aspects of medicine. Accordingly, Evans et al. call for explicit teaching of the epistemological bases of medicine, explaining that “physicians are largely unaware of the power such models exert on their thinking and behavior… because the dominant models are not necessarily made explicit”.^36^ An approach that is both reflective and intentionally cultivates discussion of underlying epistemologies may help students understand and integrate what they perceive as incongruous domains of the humanistic and biophysical aspects of illness.^47^

The importance of reflection as a technique to address epistemic cognition is emphasized throughout the medical education literature. In their critical narrative review of reflection in medical education research, Ng, Kinsella, Friesen, and Hodges frame reflection as an epistemology of practice.^50^ As such, reflective practice offers opportunities for practitioners and students to question reframe and re-evaluate their knowledge, experience, actions and decisions. A reflective approach positions practice as a site for developing new knowledge and learning to navigate uncertainty, engaging with a variety of sources of knowledge including the tacit and experiential. Medical educators commonly use reflection to foster critical social inquiry and knowledge-building amidst uncertainty.^50^ Facilitating reflection on examples from biomedical science research can help students develop sophisticated beliefs about certainty and source of scientific knowledge. Narrative reflection on patient interactions can help students to recognize complexity and uncertainty in patient-care situations and the subjective, culturally infused nature of disease.

Reflection on patient interactions may help to challenge beliefs about uncertainty, complexity, and multiple perspectives in medicine. Also, engaging medical students in reflection and constructive debate about cultural diversity can help them understand “that dealing with subjectivity, diversity, ambiguity and uncertainty is inseparable from the personal dimension of medicine as moral enterprise.”^21^

The implications for educators understanding how medical students think are broad.  For example, students with more complex epistemological approaches may be more open to utilizing broader illness frameworks (such as the biopsychosocial model) than students with more concrete epistemic tendencies. Given initiatives from institutions and organizations involved in medical education to increase the capacity of physicians to utilize social and psychological factors in the clinical encounter,^64^^,^^65^^,^^66^ developing greater understanding of those potential epistemic factors is highly important.

In order to achieve goals of integrating epistemology into medical education and practice, institutional support, faculty buy-in, and professional development will be necessary. Additionally, educators and researchers must consider how prominent epistemological perspectives in medical education, such as prioritization of research knowledge above experiential knowledge or reductionist approaches to assessment, may influence or impede implementation of new approaches to medical education.

## Conclusions

Clearly, aspects of epistemic cognition are addressed in the medical literature in different ways. Developing new research instruments and protocols can enhance the validity of studies, while other qualitative methods, such as narrative analysis and observation of participants in naturalistic settings, may help researchers examine the complexities of epistemology. Along with methodological developments, substantive areas for future research arise from our review. These include questions about the interrelatedness of different frameworks (e.g. how developmental frameworks of epistemic cognition^13^^,^^14^^,^^29^ interface with the biopsychosocial/biomedical model) and whether epistemic cognition constitutes context specific beliefs or more stable traits. A fine-grained approach to studying epistemic cognition in medical students and physicians could reveal specific aspects of the educational and clinical context that trigger certain epistemological resources.^10^^,^^54^ Researchers should take into consideration epistemic aims and epistemic values that contribute to nuances in individuals’ conceptions and behavior.^5^ Also, while few studies examine epistemic cognition in other health fields, there is evidence that sophistication of epistemic beliefs with experience occurs across professions.^67^^,^^68^ Studies comparing epistemic orientations including biomedical or biopsychosocial orientations^68^ or patterns of knowing for practice among medical, nursing and allied health fields^69^^,^^70^^,^^71^  could provide further insights for both medical and inter-professional education.

While epistemic cognition is assumed to underlie tolerance of ambiguity and uncertainty, and studies of medical epistemology suggest they do,^30^^,^^36^ several questions remain unanswered. Does the level of sophistication of epistemic cognition as a whole^12^^,^^13^^,^^14^^,^^29^ and within the different dimensions of epistemic cognition^6^^,^^8^^,^^9^ influence stress students or physicians experience when confronted with ambiguous or uncertain situations, and if so how? Does the decrease in physicians’ anxiety over time relate to development of more sophisticated epistemic beliefs or other coping mechanisms? Does stress in medical contexts serve to stunt or drive forward epistemological development? Examining these relationships could bring new insights to the ambiguity tolerance literature and contribute to educational interventions designed to facilitate management of uncertainty.

The relationship between epistemic cognition and patient outcomes represents a critical question for extending these discussions beyond the academic. While some studies have associated physician uncertainty with increased hospital admissions,^72^ excessive testing,^73^ and increased morbidity and mortality,^74^ it is unclear whether epistemic orientation toward uncertainty mediates these relationships. Additionally, exploring how epistemic cognition interfaces with adaptive expertise could provide important insights for educating physicians, considering that contextualized knowledge, awareness of uncertainty, and innovative thinking have been found to distinguish experts from novices.^75^^,^^76^ Further research is needed to determine what kinds of educational resources and supports are necessary to enhance students’ and physicians’ epistemic cognition.

To move this field of research forward, it is necessary to further develop coherent theoretical frameworks to conceptualize and facilitate critical examination of epistemic cognition in physicians and medical students. Incorporating humanism into medical education and fostering epistemic cognition appear to be interdependent endeavors with great potential to advance mutually an integrative and humane approach to medical practice.

### Acknowledgements

This project was made possible with a grant from the Arnold P. Gold Foundation. We would like to thank Mark MacEachern for consultation and excellent work on the literature search process.

### Conflict of Interest

The authors declare that they have no conflict of interest.

## Supplementary material

Supplementary file 1 Appendix 1. Studies selected for review
