# Supplementary material for: Epistemic cognition in medical education: a literature review
Source: Int J Med Educ. 2017 Jan 7;8:1–12. doi: 10.5116/ijme.5849.bfce (PMC5222678; doi:10.5116/ijme.5849.bfce)
Supplement: Supplementary file 1 — Appendix 1. Studies selected for review [file ijme-8-1-S1.pdf]

Appendix 1

Studies selected for review

First Author	Year	Country	Participants	Methodology	Instrument/Data Source
Borgstrom	2013	England	123 M4 Students	Qualitative	Essays, Course materials, Interviews
Brennan	2010	UK	31 Residents	Qualitative	Interviews, Audio diary
Clandinin	2008	Canada	4 Residents, Family Medicine	Qualitative	Written charts, Reflective group dialog
Cristancho	2013	Canada	7 Physicians, Surgery	Qualitative	Interviews
de Camargo	2002	Brazil	14 Physicians, Internal Medicine	Qualitative	Interviews
De Valck	2001	Belgium	88 Belgian and 434 Dutch Medical Students	Quantitative	Ideal Physician Questionnaire[38]
DeForge	1991	USA	59 Residents, Family Medicine	Quantitative	Tolerance for Ambiguity Scale[49]
Dogra	2007	UK	61 Medical Teachers, Administrators, Policy Makers, Researchers, Medical Students, Patients	Qualitative	Interviews
Evans	2009	USA	78 Resident and Non-Resident Physicians, Primary Care	Quantitative	Physician's Belief Scale,[77] Physicians', Reactions to Uncertainty Scale[78]
Evans	2012	USA	89 M3 Students	Quantitative	Physician's Belief Scale,[77] Physicians', Reactions to Uncertainty Scale[78]
Fyrenius	2007	Sweden	16 M1 Students	Qualitative	Interviews
Geller	1990	USA	386 M1, M2, M3, M4 Students	Quantitative	Tolerance for Ambiguity Scale,[49] Quantitative survey on beliefs on ambiguous situations
Gerrity	1992	USA	428 Physician Faculty, Internal Medicine, Family Medicine, Surgery	Mixed Methods	Physicians' Reactions to Uncertainty Scale,[78] Qualitative questionnaire
Gordon	2012	Australia	20 Physicians, Multiple Fields	Qualitative	Interviews
Klaczynski	1994	USA	43 M1 Students, M4 Students, 33 Graduate Students	Mixed Methods	Developmental Task Questionnaire, Everyday Problem-Solving Instrument, Selection Task, Interpretive Style Dilemmas, Interview
Knight	2006	UK	15 M2 Students	Qualitative	Interview based on published protocols[79,13]
Lingard	2003	Canada	11 M3 Students, 10 Faculty, Pediatrics	Qualitative	Interviews, Case Presentations
Lonka	1996	Finland	175 M1, M4 Students, and Psychology Graduate Students	Mixed Methods	Approaches to Studying Inventory,[80] Inventory of Learning Styles,[81] Perry's Dualism Scale,[82] Essay
Marambe	2007	Sri Lanka	288 M1 Students in Traditional and Active Learning Curricula	Quantitative	Adyayana Rata Prakasha Malawa (ARPM), adapted Sinhala version of the Inventory of Learning Styles,[81] Learning Strategy, Conceptions of Learning and Learning Orientation
First Author	Year	Country	Participants	Methodology	Instrument/Data Source
Nevalainen	2010	Finland	22 M3, M4 Students	Qualitative	Reflective writing
Roex	2009	Belgium	29 Resident and Non-Resident Physicians, General Practice	Qualitative	Focus groups, Discussion of ill-structured problems
Sherrill	2001	USA	87 MD/MBA Students, 115 MD Students	Qualitative	Tolerance for Ambiguity Scale[49]
Simpson	1986	USA	27 M3 Students, Residents, Physician Faculty, Family Medicine	Qualitative	Measure of Intellectual Development[83]
Sobal	1991	USA	171 M1 Students	Quantitative	Uncertainty scale based on Tolerance for Ambiguity Scale[49]
Stolper	2008	Netherlands	28 Physicians, Family Medicine	Qualitative	Focus groups, Scenario discussion
Timmermans	2001	USA	17 Residents, Pediatrics	Qualitative	Interviews
Weissenstein	2014	Germany	593 M1, M2, M3, M4 Students, Physicians, Family Medicine	Quantitative	Inventory for measuring ambiguity tolerance[84]
